# Genetic Diversity of *Sogatella furcifera* (Hemiptera: Delphacidae) in China Detected by Inter-Simple Sequence Repeats

**DOI:** 10.1093/jisesa/ieu095

**Published:** 2014-01-01

**Authors:** Jia-Nan Xie, Jian-Jun Guo, Dao-Chao Jin, Xue-Jian Wang

**Affiliations:** The Provincial Key Laboratory for Agricultural Pest Management of Mountainous Region, Institute of Entomology, Guizhou University, Guiyang, Guizhou 550025, China

**Keywords:** white-backed planthopper, ISSR-PCR, genetic diversity, geographic population

## Abstract

The white-backed planthopper (WBPH), *Sogatella furcifera* (Horváth) is a serious pest causing grievous damage to rice plants. In the present study, inter-simple sequence repeats were employed to investigate the genetic diversity of 108 samples from 27 WBPH geographic populations in China. Ten primers were screened out with 147 amplified bands, average percentage of polymorphic bands, polymorphic information content, and marker index were 78.9, 0.456, and 6.753% respectively. The results indicated that genetic diversity was different among populations, but genetic variation was as low as 0.2% among the populations and as high as 99.8% within the same geographic population. Among the examined WBPH populations, genetic distances were weakly correlated to geographic distance, and there was no correlation between genetic identity and elevation. Cluster analysis showed that the 27 WBPH populations studied could be lumped into four clusters, with which the results of principal coordinate analysis (were almost consistent. In conclusion, the molecular genetic data demonstrated that the region consisting of Yunnan, Guizhou, Guangdong, and Guangxi was the first landing area of WBPH in its migrating process from overwintering sites to China.

The white-backed planthopper (WBPH), *Sogatella furcifera* (Horváth) (Hemiptera: Delphacidae), is a serious pest of rice worldwide in distribution ( Kuoh et al. 1983 , Qin and Ye 2003 ). It causes grievous damage to rice plants, such as reduction of vigor, tillering delay, yellowing of leaves, and shriveling of grains ( Khan and Saxena 1984 ), even causing death as a consequence of piercing-sucking and egg-laying ( Pathak 1968 ). In recent years, WBPH has caused especially great devastation and even total crop loss with the change of rice planting system from the 1950s ( Zhao et al. 2011 ). This has generated a lot of research on WBPH, including topics such as biological characteristics, migration patterns, outbreak patterns, and population dynamics ( Shen et al. 2003 ), but there has been little research about population genetic structure and genetic differentiation.

High-altitude aerial netting, ship-catching, recapture of marked insects, dissection of female ovaries, radar monitoring, and trajectory analysis studies have found that the original source of WBPH in China is the Indo-China Peninsula aided by the southwest monsoon ( Hu and Liu 1981 ). However, due to its nocturnal migration habits and tiny body, study on WBPH migration, and especially regional migration is still difficult and obscure. With the development of molecular biology, more and more molecular techniques are incorporated into diverse research fields, and this also provides new methods to analyze migration of WBPH by examining population genetic structure and genetic differentiation ( Liu et al. 2010 ).

Inter-simple sequence repeats (ISSRs) is a technique of molecular markers, which was developed by Zietkiewicz et al. (1994) and has been widely used in studies on genetic diversity of geographic populations ( Fang 2006 , Chu et al *.* 2008 , Weng et al. 2008 ) due to its advantages. Such advantages include easy synthesis, better universality of the primers compared with simple sequence repeat (SSR); more information and higher polymorphism of the amplified products compared with randomly amplifiled polymorphic DNA (RAPD); and a similar veracity than restriction fragment length polymorphism (RFLP), but with higher stability and detectability ( Pu et al. 2007 ).

Deepening our knowledge on WBPH, including that on genetic structure, genetic diversity, and migration, is important for the development of integrated control strategies and to reduce economic losses. In this study, ISSRs were employed to investigate the genetic diversity of 108 samples from 27 WBPH geographic populations. This study was undertaken to enrich the genetic and migratory knowledge on WBPH, which could provide information to develop regional strategies of integrated control.

## Materials and Methods

### 

#### Sampling

**Fig. 1.**
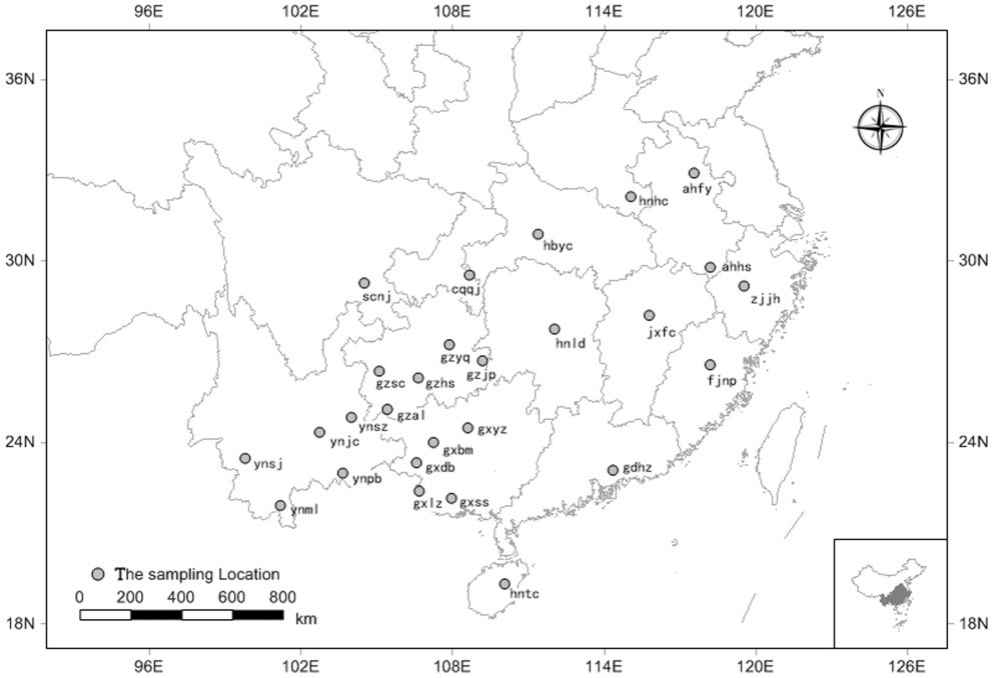
Collection sites for 27 geographic populations of WBPH in China. The abbreviations for the 27 WBPH geographic populations were as fallows: ahfy for Fengyang, Anhui; ahhs for Huangshan, Anhui; cqqj for Qianjiang, Chongqing; fjnp for Nanping, Fujian; gdhz for Huizhou, Guangdong; gxbm for Bama, Guangxi; gxdb for Debao, Guangxi; gxlz for Longzhou, Guangxi; gxss for Shangsi, Guangxi; gxyz for Yizhou, Guangxi; gzal for Anlong, Guizhou; gzhs for Huishui, Guizhou; gzjp for Jinping, Guizhou; gzsc for Shuicheng, Guizhou; gzyq for Yuqing, Guizhou; hntc for Tunchang, Hainan; hnhc for Huangchuan, Henan; hbyc for Yichang, Hubei; hnld for Loudi, Hunan; jxfc for Fengcheng, Jiangxi; scnj for Neijiang, Sichuan; ynjc for Jiangchuan, Yunnan; ynml for Menglun, Yunnan; ynpb for Pingbian, Yunnan; ynsj for Shuangjiang, Yunnan; ynsz for Shizong, Yunnan; and zjjh for Jinhua, Zhejiang.

One hundred and eight samples (macropterous; four samples at each location) were collected from 27 locations of rice-planting areas where WBPH causes serious damage in 14 provinces of China ( Fig. 1 ). The collecting period was from April to June [Collection time and capture of long-winged individuals were established to make sure that the specimens collected were migrants ( Wang et al. 1982 , Zhu et al. 2001 )], These samples were preserved in 100% alcohol at −20°C until use. Information about longitude, latitude, and altitude was recorded.

## Methods

### 

#### DNA Extraction

Total genomic DNA was extracted from WBPH using the modified sodium-dodecyl sulphate (SDS) method ( Wang et al. 2001 ). DNA concentration and quality were measured with a SmartSpec Plus Spectrophotometer (Bio-Rad, CA, USA). The extracted DNA was stored at −20°C until needed.

#### ISSR-PCR Amplification

One hundred primers were selected from the ISSR primer set developed by Michael Smith Laboratories at the University of British Columbia (UBC)and synthesized by Sangon (Shanghai, China). Ten primers that produced bright, clear, and repeatable bands were screened for further studies ( Table 1 ).

**Table 1.** ISSR primers and the polymorphism of their PCR products for 27 WBPH Chinese populations

Sequence	Annealing temperature (°C)	Number of bands scored	Number of polymorphic bands	PPB (%)	PIC	MI
(GA) _8_ C	52.3	25	21	84	0.494	12.35
(GA) _8_ YG	54	11	10	90.1	0.496	5.456
(AG) _8_ YT	52.3	13	12	92.3	0.474	6.162
(AG) _8_ TA	54	12	9	75	0.479	5.748
(CA) _8_ A	51.7	13	9	69.2	0.41	5.33
(CA) _8_ RT	51.2	17	14	82.4	0.497	8.449
(AC) _8_ YG	54	14	9	64.3	0.373	5.222
(AC) _8_ TC	53	16	11	68.8	0.433	6.928
(AC) _8_ ATT	52.3	11	9	81.8	0.422	4.642
(AC) _8_ AG	51	15	12	80	0.483	7.245
Average		14.7	11.6	78.9	0.456	6.753

Notes: Y = 1/2(C,T); R = 1/2(A,G).

Optimized reaction system of ISSR-PCR: each PCR amplification reaction mixture comprised 50 ng of DNA, 2 μl of reaction buffer (10 mmol/L Tris-HCl; 50 mmol/L KCl), 1.5 mmol/L Mg ^2+^ , 250 μmol/L dNTPs, 0.9 μmol/L primer, and 1 U Taq DNA Polymerase (TaKaRa, Dalian, China), and addition of ddH _2_ O to make a total volume of 20 μl.

Amplification was performed in an iCycler Thermal Cycler (Bio-Rad) under the following conditions: an initial denaturation of 94°C for 4 min, followed by 40 cycles of denaturing at 94°C for 1 min, annealing for 1 min (temperature depending on primers used), extension at 72°C for 2 min, and a final extension at 72°C for 10 min. The PCR products were separated on 2% agarose gels in 0.5× TBE buffer and detected by staining with GoldView I (Solarbio, Beijing, China). Bands sizes were compared with 100 bp DNA ladder marker (TaKaRa), and the intensities of bands were determined by spectrophotometry using ImageQuantity One (Bio-Rad).

#### Data Analysis

Band patterns were recorded with a Universal Hood II Imaging System (Bio-Rad). Amplified products were scored from the gel images according to presence or absence of bands (1 = present and repeatable; 0 = absent or unrepeatable; and 9 = not amplified, missing value). Thus a matrix (1, 0) was obtained.

Because ISSR markers are dominantly inherited, each band was assumed to represent the phenotype at a single biallelic locus ( Williams et al. 1990 ). Number of bands scored, number of polymorphic bands, and percentage of polymorphic bands (PPB) were calculated with Excel according to the (0, 1) matrix mentioned earlier. Polymorphic information content (PIC, $\text{PIC}=1-\sum_{i=1}^{m}P_{i}^{2}-\sum_{i=1}^{m-1}\sum_{j=i+1}^{m}2P_{i}^{2}P_{j}^{2}$ , $P_{i_{}}$ and $P_{j}$ are allele frequencies of NO. i and NO. j , m is the number of allele) and marker index (MI, *MI* = Allele × PIC, Allele is the number of allele) of each primer were calculated according to the information in the matrix ( He et al. 2010 , Yu and Liu 2010 ).

Effective alleles (Ne), Nei’s gene diversity ( *H* ), and Shannon’s index ( *I* ) within a population and genetic distance and genetic identity among populations were calculated using POPGENE Version 1.31 software ( Yeh et al. 1999 ) according to the (0, 1) matrix. Analysis of molecular variance (AMOVA) was analyzed according to genetic distances using Arlequin Version 3.11 software ( Excoffier et al. 2005 ).

Cluster analysis [unweighted pair group method with arithmetic mean (UPGMA)] and principal coordinate analysis (PCOA) were performed according to genetic diversity. Mantel tests were performed between genetic diversity and cluster analysis. All analyses mentioned earlier were performed with NTSYS-pc 2.10e software ( Rohlf 2000 ).

Mantel tests were performed with TFPGA Version 1.3 ( Miller 1997 ) to examine significant relationships between genetic and geographic distances, and between genetic identity and elevation differences.

## Results

### 

#### ISSR Profile

According to the brightness, clarity, and repeatability of bands, 10 primers ( Table 1 ) were selected from the screening of a set of 100 primers. The 10 primers amplified a total of 147 bands that were bright, clear, and repeatable. The number of bands produced by individual primer ranged from 11 to 25 with an average of 14.7. PPBs were from 64.3 to 92.3% with an average of 78.9% (116 bands). PICs were in a range of 0.373–0.497; MIs were in a range of 4.642–12.350. The levels of polymorphism and the information generated here confirmed that ISSR primers were valuable for the study of WBPH genetic diversity ( Yu and Liu 2010 ).

#### WBPH Genetic Diversity

Based on the matrix of amplified fragments, the genetic diversity indexes calculated using POPGENE Version 1.31 are shown in Table 2 . PPB values ranged from 42.18 (ahhs) to 67.35% (scnj), effective alleles (Ne) values from 1.2966 ± 0.3667 (ahhs) to 1.5075 ± 0.3891 (scnj), Nei’s gene diversity ( *H* ) values from 0.1718 ± 0.2049 (ahhs) to 0.2849 ± 0.2052 (scnj), and Shannon’s index ( *I* ) values from 0.2514 ± 0.2977 (ahhs) to 0.4125 ± 0.2930 (scnj). Results of *H* , *I* values indicated that genetic diversities were different among populations:

**Table 2.** Genetic diversity of Chinese WBPH geographic populations

Geographic population list	Number of polymorphic bands	PPB (%)	Effective alleles (Ne)	Nei’s gene diversity ( *H* )	Shannon’s index ( *I* )
gxyz	85	57.82	1.4286 ± 0.3952	0.2423 ± 0.2126	0.3519 ± 0.3053
gxss	97	65.99	1.4558 ± 0.3556	0.2662 ± 0.1964	0.3906 ± 0.2849
gxlz	86	58.5	1.4463 ± 0.4062	0.2491 ± 0.2158	0.3601 ± 0.3083
gxdb	83	56.46	1.3986 ± 0.3756	0.2304 ± 0.2073	0.3371 ± 0.3002
gxbm	89	60.54	1.4422 ± 0.3869	0.2517 ± 0.2090	0.3663 ± 0.3005
ynpb	85	57.82	1.4095 ± 0.3762	0.2364 ± 0.2070	0.3456 ± 0.2995
ynjc	84	57.14	1.4299 ± 0.4016	0.2415 ± 0.2148	0.3498 ± 0.3078
ynml	83	56.46	1.4095 ± 0.3870	0.2338 ± 0.2106	0.3406 ± 0.3036
ynsj	93	63.27	1.4531 ± 0.3755	0.2602 ± 0.2040	0.3798 ± 0.2942
ynsz	83	56.46	1.4340 ± 0.4104	0.2415 ± 0.2178	0.3487 ± 0.3110
gzal	90	61.22	1.4490 ± 0.3879	0.2551 ± 0.2089	0.3710 ± 0.3002
gzsc	84	57.14	1.4327 ± 0.4041	0.2423 ± 0.2156	0.3507 ± 0.3086
gzhs	84	57.14	1.4136 ± 0.3858	0.2364 ± 0.2100	0.3445 ± 0.3028
gzyq	76	51.7	1.4000 ± 0.4135	0.2219 ± 0.2198	0.3201 ± 0.3140
gzjp	88	59.86	1.4218 ± 0.3724	0.2440 ± 0.2050	0.3571 ± 0.2968
cqqj	82	55.78	1.4190 ± 0.4016	0.2355 ± 0.2153	0.3413 ± 0.3086
hnld	90	61.22	1.4517 ± 0.3904	0.2560 ± 0.2096	0.3719 ± 0.3009
jxfc	94	63.95	1.4653 ± 0.3812	0.2653 ± 0.2053	0.3863 ± 0.2951
fjnp	91	61.9	1.4449 ± 0.3787	0.2551 ± 0.2058	0.3721 ± 0.2967
zjjh	98	66.67	1.5007 ± 0.3892	0.2815 ± 0.2058	0.4078 ± 0.2940
hbyc	88	59.86	1.4354 ± 0.3858	0.2483 ± 0.2090	0.3616 ± 0.3008
scnj	99	67.35	1.5075 ± 0.3891	0.2849 ± 0.2052	0.4125 ± 0.2930
gdhz	95	64.63	1.4721 ± 0.3817	0.2687 ± 0.2050	0.3910 ± 0.2945
hntc	99	67.35	1.4993 ± 0.3828	0.2823 ± 0.2033	0.4099 ± 0.2910
hnhc	84	57.14	1.4000 ± 0.3716	0.2321 ± 0.2059	0.3400 ± 0.2986
ahhs	62	42.18	1.2966 ± 0.3667	0.1718 ± 0.2049	0.2514 ± 0.2977
ahfy	63	42.86	1.3116 ± 0.3812	0.1777 ± 0.2094	0.2588 ± 0.3025

Note: Values of traits are mean ± SD (standard deviation).

AMOVA indicated that 99.8% of genetic variability was within the same geographic population, and only 0.2% among different geographic populations ( Table 3 ). A random permutation test showed that variation of populations was not significant ( *P* = 0.152, > 0.05), which was confirmed by *F*_ST_ values (0.00199).

**Table 3.** AMOVA in 27 populations of WBPH using 147 ISSR markers

Source of variation	df	Sum of squares	Variance components	Percentage of variation (%)	*P* -value
Among populations	26	3.502	0.00099	0.2	0.152
Within a population	9861	4900.637	0.49598	99.8	<0.001

#### Cluster and Correlation Analysis

Results of a Mantel test on genetic diversity and a cluster analysis yielded a correlation index of 0.75, which indicated that the cluster was representative of the genetic relationship among populations ( Kong et al. 2004 ).

**Fig. 2.**
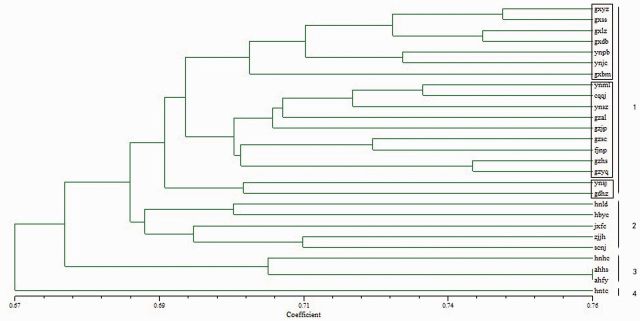
Cluster analysis of the 27 WBPH Chinese populations based on genetic diversity (1) (UPGMA).

**Fig. 3.**
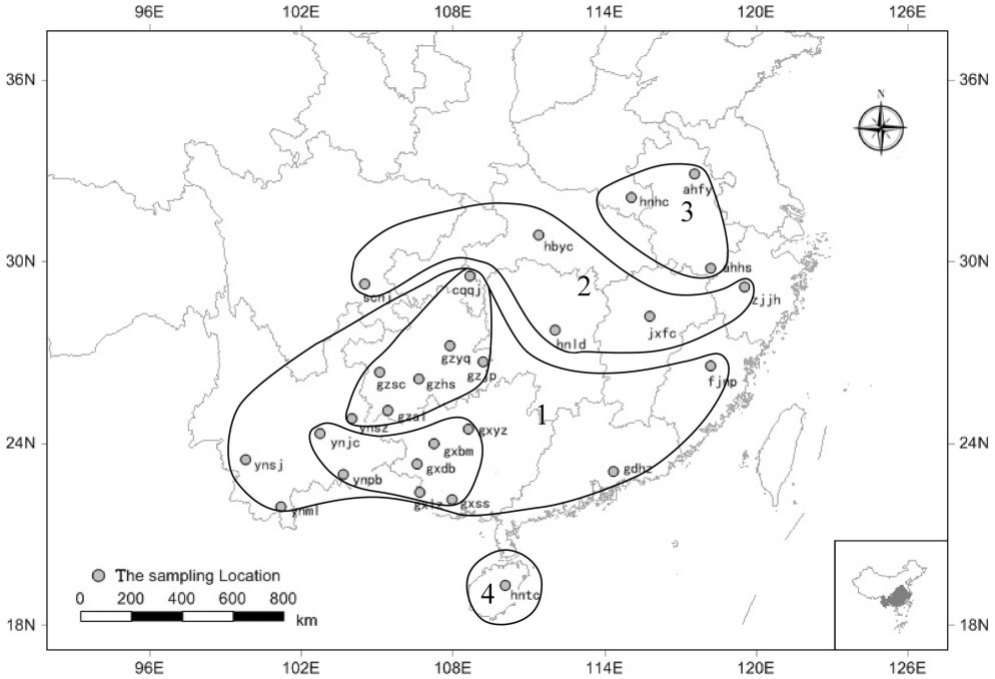
Cluster analysis of the 27 WBPH Chinese populations based on genetic diversity (2).

Results demonstrated that there is genetic differentiation among the 27 geographic populations of WBPH. The tree of cluster analysis ( Figs. 2 and 3 ) grouped populations into four clusters, i.e., the first one included 18 populations from Guangxi (gxyz, gxss, gxlz, gxdb, and gxbm), Yunnan (ynpb, ynjc, ynml, ynsz, and ynsj), Guizhou (gzal, gzsc, gzhs, gzyq, and gzjp), Chongqing (cqqj), Fujian (fjnp), and Guangdong (gdhz); the second included 5 populations from Hunan (hnld), Hubei (hbyc), Sichuan (scnj), Jiangxi (jxfc), and Zhejiang (zjjh); the third one included 3 populations from Henan (hnhc) and Anhui (ahhs and ahfy); and the fourth one included 1 population from Hainan (hntc). It also revealed that genetic differentiation was correlated to geographic distance either between the clusters or within the same cluster.

**Fig. 4.**
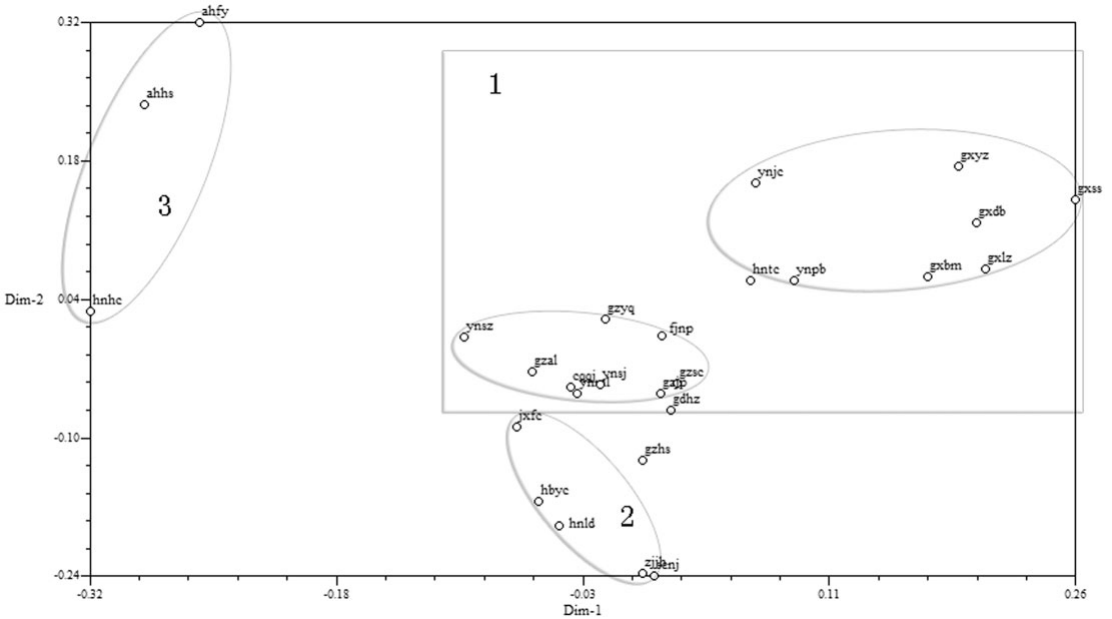
PCOA of the 27 geographic populations of WBPH based on genetic diversity.

Results of PCOA on 27 geographic populations showed that the contributions of the anterior three principal coordinates were 32.13, 29.25, 17.58, respectively, and the accumulation was 78.96 ( Wei et al. 2012 ). According to Fig. 4 , 27 geographic populations were divided into three groups. Group 2 included 6 units (populations): jxfc, gzhs, hbyc, hnld, zjjh, and scnj; Group 3 included 3 units: ahfy, ahhs, and hnhc; Group 1 was divided into 2 small groups, of which the first included 8 units: gxss, gxyz, gxdb, gxlz, gxbm, ynjc, ynpb, and hntc, and the second 10 units: ynsz, ynml, gzal, gzyq, gzsc, gzjp, cqqj, ynsj, fjnp, and gdhz. In the third principal coordinate, hntc was far from the other two populations; gzhs was far from Group 2; and gdhz was far from the second small group in Group 1.

**Fig. 5.**
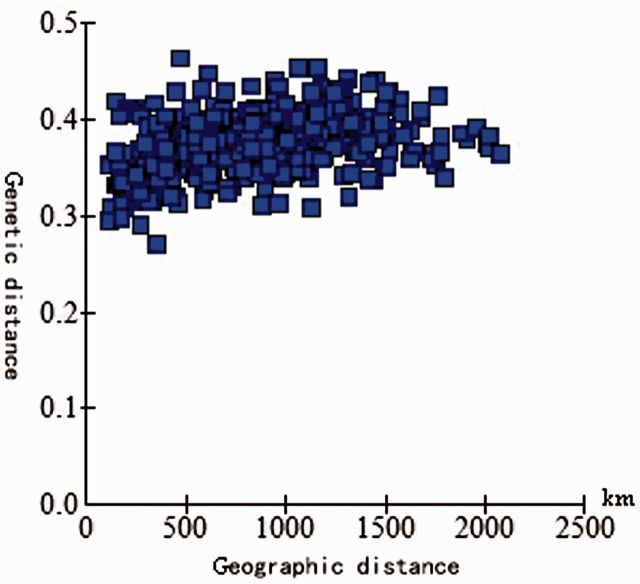
Correlation analysis between genetic and geographic distances.

**Fig. 6.**
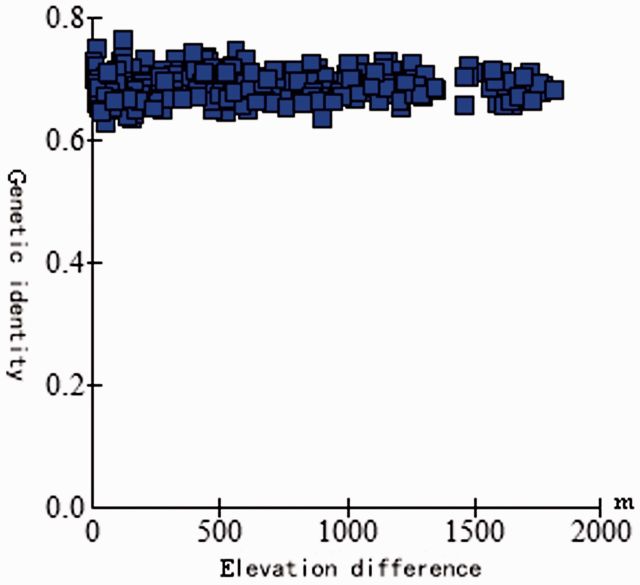
Correlation analysis between genetic identity and elevation differences.

The results of PCOA and cluster analysis were almost consistent. (Differences existed only for the ynsj and gzhs populations). Correlation analysis of 27 WBPH geographic populations in China showed that genetic and geographic distances were weakly correlated [Mantel test ( Mantel 1967 ): *r* = 0.3216, *P* = 0.001] ( Fig. 5 ; Liu et al. 2013 ). The distance between ynsj and zjjh populations was the longest, and genetic distances was 0.3630. The genetic identity was not correlated with elevation differences (Mantel test: *r* = 0.0857, *P* = 0.164; Fig. 6 ). The largest elevation difference (1,812 m) was between ynsz and ahfy, and the genetic identity index was 0.6820.

## Discussion

Previous research has shown that WBPH can survive winter at only a few places in the south part of the Yunnan, Guangxi, and Hainan provinces ( Qin and Ye 2003 ). The annual early WBPH migration source in China is mostly the Indo-China Peninsula aided by the southwestern monsoon ( Hu and Liu 1981 ). So the corollary of the migratory path would be more representative for studies based on analysis of migrant WBPH specimens. The specimens used in this study were collected during April to June. At that period, there were lots of specimens in the fields and most were landing following migration (macropterous). Twenty-seven locations were chosen in rice-planting areas, where rice plants were severely damaged by WBPH ( Qin and Ye 2003 ). Four sampling sites were chosen at each location and 50 specimens were collected at each sampling site, then 1 specimen was randomly chosen for extraction.

In this study, experimental procedures were standardized to control the effect of confounding factors that were observed in previous studies ( Xie et al. 2014 ). A number of bright, clear, and repeatable bands were amplified with the selected 10 primers from 27 populations of WBPH. PPB, PIC, and MI showed that the primers could be used to study genetic diversity of WBPH and that more reliable information could be obtained from the amplified products (contrasted with other 90 primers).

Genetic diversity of different geographic populations was different, but differences were small ( Table 2 ); AMOVA showed that genetic variation among different geographic populations (0.2%) was much smaller than that within a population (99.8%). *F*_ST_ values revealed no obvious differentiation. Mantel test results showed that the correlation between geographic and genetic distance, and genetic diversity and elevation were both weak. All results above showed that broad gene exchange is occurring among sampled populations. The possible reason is likely the long-distance migratory capability of WBPH ( Lv et al. 2013 ).

Based on genetic differentiation of the 27 geographic populations of WBPH, cluster analysis revealed the existence of four tightly linked groups. In the first cluster, populations from Guangxi and ynpb, ynjc from Yunnan were grouped together as a subcluster at the top of the tree. This can be taken as evidence of a close genetic relationship with minor genetic difference, and suggests that WBPH collected in different localities originate from source areas belonging to a large population, a conclusion that coincides with those of similar studies ( Shen et al. 2011a ). Populations from Guizhou and Chongqing were gathered with another two populations from Yunnan (ynml and ynsz), indicating that the original populations of the former two provinces may partially be from Yunnan. Those five populations from Yunnan separated by small geographic distance were not grouped together, which indicates that insects originate from different sources. Those results earlier suggest that southwest part of Yunnan and Guizhou are on one migration pathway of WBPH, while southeast part of Yunnan and Guangxi are on another pathway. The structure of populations is caused by different insect sources and migration pathways, which is also a conclusion reached by some other studies ( Shen et al. 2011b ).

These eight populations on the second and third clusters were located northeast of the first cluster, a result that may be produced by WBPH’s routes of migration northeastward with southwest monsoon ( Hu and Liu 1981 ). The population of hntc from Hainan alone formed the fourth cluster at the base of the tree, indicating that hntc has a weak genetic relationship with the other 26 populations, and WBPH from Hainan has no obvious relationship with the other populations, which shows that Hainan is seldom the insect source region of other districts in China.

Migration of WBPH from Indo-China Peninsula to China depends on the southwest monsoon ( Hu and Liu 1981 ). In view of the first studies on WBPH in China ( Qin and Ye 2003 ) and the Results and Discussion in this article, it can be speculated that the region constituted by Yunnan, Guizhou, Guangdong, and Guangxi is the first landing region of WBPH in its migrating process from Indo-China Peninsula or Southeast Asia to China under effects of the southwest monsoon; and with the monsoon becoming stronger, the migration area becomes larger, i.e., WBPH lands in Yunnan, Guizhou, Guangdong, and Guangxi at the beginning, and then moves northward and northeastward to Hunan, Hubei, Sichuan, Jiangxi, and Zhejiang, further to Henan, Anhui, and Jiangsu, and even to more distant locations ( Hua et al. 2003 , Diao et al. 2012 ).

In-depth knowledge of migration pathways and sources is essential for the development of WBPH area wide control strategies. According to our conclusions, some suggestions are given as follows: 1) strengthen international cooperation and communication for management of insect populations in the source region and for production of accurate yearly premigration forecasts; 2) strengthen regional cooperation between western and eastern China, to detect seasonal initial landing of WBPH, and then issue timely forecasts to secondary seasonal landing sites; 3) strengthen application of control measures of WBPH in Guangxi, Guangdong, Yunnan, Guizhou, to decrease its quantity in fields, and therefore reduce the size of remigrant populations at its Chinese initial seasonal source to secondary seasonal migrating sites. Given the migrant nature of this important pest, best management results can only be obtained through cooperative area-wide coordinated control programs.
